# Performance of multimodal large language models on image‐based surgical anatomy, anatomical pathology, and radiology questions

**DOI:** 10.1002/ase.70256

**Published:** 2026-05-13

**Authors:** Ming Lu, Josiah Cheng, Vinod Gopalan

**Affiliations:** ^1^ Medical Education, School of Medicine and Dentistry Griffith University Southport Queensland Australia; ^2^ Gold Coast University Hospital Southport Queensland Australia; ^3^ Logan Hospital Meadowbrook Queensland Australia

**Keywords:** anatomy, assessment, histopathology, large language models, medical education, multimodal AI, radiology

## Abstract

Multimodal large language models (LLMs) are now deeply integrated into medical education and widely used by medical students, yet it remains unclear whether current models possess the accuracy and reliability needed to support image‐based learning. We evaluated four state‐of‐the‐art multimodal LLMs (ChatGPT‐5.1, Gemini‐2.5, Grok‐4, Claude Sonnet‐4.5) on 208 image‐based examination questions from a Doctor of Medicine program, spanning anatomical pathology (histopathology; 47.6%), radiology (31.7%), and surgical anatomy (20.7%). To isolate visual reasoning, all items were presented in image‐only form with contextual information removed. Items covered seven organ systems, included both constructed‐response and selected‐response formats, and were categorized as recognition‐only or recognition‐plus‐reasoning. ChatGPT‐5.1 achieved the highest accuracy (75.5%; 95% CI [69.2–80.8]), followed by Gemini‐2.5 (59.6%; 95% CI [52.8–66.1]), Claude Sonnet‐4.5 (41.8%; 95% CI [35.3–48.6]), and Grok‐4 (34.6%; 95% CI [28.5–41.3]). Overall model performance differed significantly (*p* < 0.001; Cramér's *V* = 0.45). Pairwise McNemar comparisons showed significant differences between ChatGPT‐5.1 and all other models (all *p* < 0.001; effect sizes 15.9–40.9 percentage points). Subgroup analyses demonstrated a consistent hierarchy (ChatGPT > Gemini > Claude ≈ Grok) across different categories. Accuracy was uniformly higher for recognition‐only and selected‐response items. Even the best‐performing model, ChatGPT‐5.1, answered approximately one in four questions incorrectly. These findings suggest that current multimodal LLMs cannot yet replace expert teaching in image‐based learning. Their use in medical education should therefore remain supervised and critically appraised, serving as adjuncts rather than authoritative sources.

## INTRODUCTION

Since the release of ChatGPT in late 2022, large language models (LLMs) have rapidly become embedded in the routine study practices of medical students worldwide. Surveys conducted in 2025 across multiple undergraduate and postgraduate medical programs consistently indicate that between 62.9% and 87.4% of medical students now use generative artificial intelligence (AI) and LLMs to clarify difficult concepts, generate practice questions, summarize curricular content, and rehearse clinical reasoning, with roughly half to two‐thirds reporting weekly use in some cohorts.^1^, ^2^, ^3^ This swift, widespread, and largely unsupervised adoption is already reshaping how students engage with, integrate, and organize medical knowledge.

In text‐only settings, LLMs now match or surpass the performance of medical students on standardized examinations. A meta‐analytic review of 45 studies found that GPT‐4 achieved a pooled accuracy of approximately 81% on medical licensing exams, passed nearly 90% of those evaluated, and outperformed medical students in 76.5% of comparisons.^4^ In the German written medical licensing examination, GPT‐4 scored 85%, placing it as high as the 99.5th percentile among human candidates.^5^ Newer models, including GPT‐4o, report even stronger performance, achieving 90% accuracy on large sets of USMLE‐style questions, 84.2%–88.2% on the Chinese National Medical Licensing Examination, and 98.5% on Taiwan's Senior Professional and Technical Examinations for Medical Doctors, all substantially exceeding average human performance.^6^, ^7^, ^8^ Together, these findings suggest that contemporary LLMs serve as useful tools for understanding text‐based medical concepts.

Importantly, the core competencies of medical practice extend beyond what text‐based assessments alone can measure. Medicine also depends fundamentally on the ability to interpret visual information, such as identifying anatomical structures, recognizing pathological changes, and analyzing radiological patterns, all of which are explicitly emphasized in international curriculum standards.^9^, ^10^ As a result, even exceptional performance on text‐only examinations offers an incomplete and potentially misleading indication of an LLM's educational utility because it does not evaluate the visual diagnostic reasoning essential to medical training.

The advent of multimodal LLMs, which accept both text and images, has enabled the evaluation of their visual diagnostic abilities. All major frontier models, including ChatGPT, Gemini, Grok, and Claude, now include image recognition capabilities.^11^, ^12^ However, recent studies demonstrate that their image‐based performance on medical licensing and board‐style examinations remains highly variable. On USMLE‐style datasets, GPT‐4 has achieved 26.9%–88.9% accuracy, while GPT‐4o has reached 62.5%–89.5%.^13^, ^14^, ^15^ Performance is notably weaker in surgical anatomy and anatomical pathology, where accuracy may fall to 25% for gross anatomy and 50% for anatomical pathology items.^13^ Diagnostic radiology shows the widest spread. Models may score as low as 19.9% on image‐only questions, but performance rises to 80.88% when clinical context and answer options are provided.^16^

Despite these developments, high‐quality evidence on how leading multimodal LLMs perform on authentic image‐based questions from medical school examinations remains limited. Existing studies show that contemporary models can perform well on selected visual tasks and may occasionally approach medical student levels, but such success is fragile, dataset‐dependent, and consistently weaker than text‐based performance.^8^, ^13^ Several methodological limitations further constrain the applicability of current findings to medical education. Many evaluations rely on small or highly curated item sets; few disaggregate performance by modality, question type, organ system, or cognitive demand; most use single‐run outputs from a single model family; and nearly all USMLE‐style studies include clinical context, making it difficult to determine whether models rely on visual features or textual cues when generating answers.^13^, ^14^, ^15^, ^16^ These limitations restrict the usefulness of existing evidence for medical educators and medical students seeking to select, implement, or critically appraise such tools. The aim of this study was to address this evidence gap by benchmarking the performance of four state‐of‐the‐art multimodal LLMs on authentic image‐based examination questions drawn from a Doctor of Medicine (MD) program.

## METHODS

### Study design

This comparative study evaluated the performance of four contemporary multimodal LLMs using a large, expert‐reviewed bank of image‐based surgical anatomy, anatomical pathology (histopathology), and radiology questions. A repeated‐run, blinded assessment design was used to ensure a robust and reliable evaluation. The study followed the Standardized Testing and Assessment Guidelines for Evaluating Generative Artificial Intelligence Reliability (STAGER) framework to maintain transparency and methodological rigor.^17^

### Item development, validation, and classification

All images and accompanying questions were sourced from two formative examinations in the Griffith University MD program delivered across Years 1 and 2, with item difficulty aligned to summative assessment standards.

The item set included a range of medical image types, including surgical anatomy, anatomical pathology, and radiology. Surgical anatomy images comprised intraoperative views and gross specimens obtained during surgery; anatomical pathology images consisted of microscopic histopathological specimens, while radiology images were static two‐dimensional representations derived from plain radiography, ultrasound, computed tomography (CT), and magnetic resonance imaging (MRI). The item set also incorporated both selected‐response questions (multiple‐choice and true‐or‐false questions) and constructed‐response questions (short‐answer questions). Items spanned major organ systems, including the central nervous, renal, musculoskeletal, gastrointestinal, ophthalmological, endocrine, and reproductive systems. Items were further classified by cognitive demand, distinguishing recognition‐only items (direct identification of a structure or feature) from recognition‐plus‐reasoning items (requiring interpretation of morphological features to reach a diagnosis).

**FIGURE 1:**
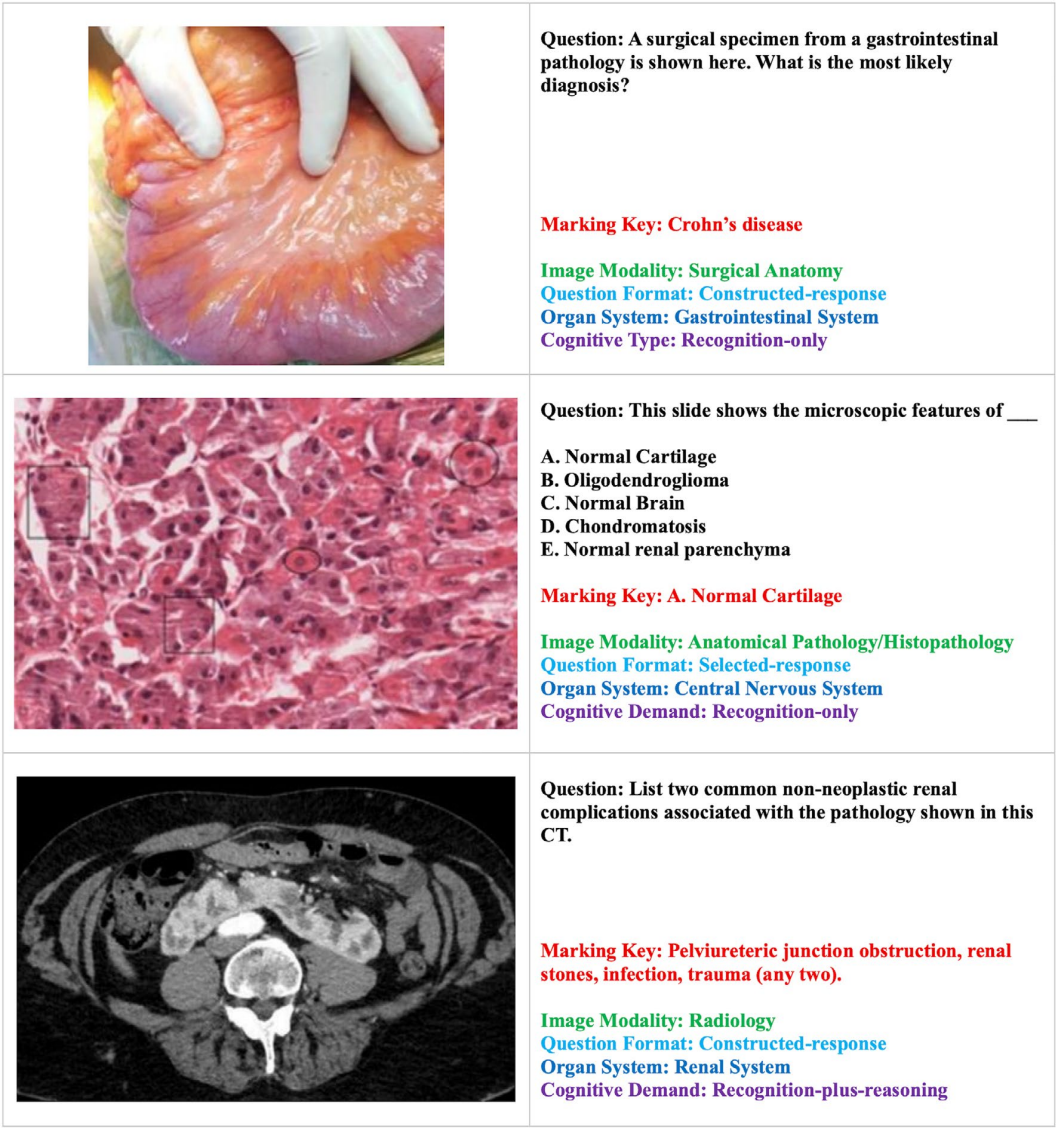
Representative examples of image‐based examination questions used in the study, illustrating typical formats across anatomical pathology (histopathology), radiology, and surgical anatomy. Images were sourced from de‐identified teaching and assessment materials within the Griffith University MD program. All images complied with IFAA ethical best‐practice guidelines and were displayed in standard clinical or anatomical orientation.

To isolate visual reasoning, all nonessential clinical or contextual information was removed, retaining only the minimal cues needed for accurate identification or diagnosis. The final question bank comprised 208 modified and content‐validated items, each independently reviewed by two medical educators (JC, VG) for image quality, prompt clarity, and single‐best‐answer validity of the pre‐existing marking key, which remained unchanged following item modification. Representative examples are shown in Figure 1. All images complied with IFAA ethical best‐practice guidelines and were displayed in standard clinical or anatomical orientation.

### Model configurations and evaluation protocol

Four multimodal LLMs, ChatGPT‐5.1 Auto (OpenAI), Gemini‐2.5 Fast (Google DeepMind), Grok‐4 Auto (xAI), and Claude Sonnet‐4.5 (Anthropic), were evaluated. All models were accessed through their paid web‐based versions to ensure consistent use of the highest‐performance configurations available at the time of testing (16 November 2025); system‐level parameters such as temperature were not user‐adjustable. All model evaluation and data extraction were performed by a single researcher (ML) using a standardized prompt:

This image is taken from a medical school examination question bank. Answer the following question using visual features of the image only. Do not use or infer any clinical or textual context, and do not access external information or search the internet. Provide only the answer, with no explanation.

Each model generated three independent responses for each item under identical testing conditions, all conducted on the same day to eliminate temporal variability. Separate chat sessions were initiated for each attempt to minimize stochastic variability, and all chat history and model memory were cleared between attempts to prevent contextual carryover.

### Scoring criterion

All responses were scored against gold‐standard marking keys developed by a panel of surgeons, anatomical pathologists, radiologists, and medical educators. The study used an all‐correct criterion, in which an item was deemed correct only if the model generated the correct answer in all three independent attempts; items with fewer than three correct answers were classified as incorrect.

### Blinded assessment

Upon completion of all evaluations, the model outputs were compiled, anonymized, and randomized. All identifiers, including model name, version, and attempt number, were removed and replaced with alphanumeric codes. Two reviewers (JC, VG), blinded to model identity and attempt order, independently evaluated each response twice against the marking keys for correctness. Disagreements were resolved through discussion, with a third adjudicator (ML) consulted when consensus could not be reached. Inter‐rater reliability was calculated using Cohen's kappa (*κ*) statistic.

### Outcome measures

The primary outcome was the proportion of questions correctly answered by each model under the all‐correct criterion. Subgroup analyses further examined performance stratified by image type, organ system, question format, and cognitive demand.

To further characterize model behavior, several exploratory metrics were calculated. Differences in item‐level performance between models were summarized using a measure derived from the discrimination index. Whereas the discrimination index is traditionally used to assess the discriminatory capacity of items across large cohorts of examinees, in this study, the same calculation (the absolute difference in accuracy between the highest‐ and lowest‐performing model for each item) was used to quantify between‐model performance difference (BMPD). BMPD was interpreted descriptively as a summary of observed differences in item‐level accuracy between models, rather than as a psychometric measure.^18^ Inter‐model variability (IMV) was evaluated by calculating the standard deviation of model accuracy across the four LLMs for each item, and model test–retest reliability was calculated using Krippendorff's alpha (nominal).^19^, ^20^

### Statistical analysis

All analyses were performed using SPSS (version 29) and Python (version 3.10), with a two‐sided significance threshold of 0.05. Given that each question was answered by all four models, the data were treated as paired nominal outcomes. Overall inter‐model differences in accuracy were evaluated using Cochran's *Q* test. The effect size for Cochran's Q was expressed using Cramér's *V*, with values <0.29 considered weak, 0.30–0.49 moderate, and >0.50 strong.^21^, ^22^ Where significant overall differences were observed, post hoc pairwise comparisons were performed using McNemar tests, and effect sizes were reported as the absolute difference in paired proportions, interpreted using conventional benchmarks (<5% small, 5%–10% moderate, >10% large differences).^23^ BMPD and IMV values were reported using means and standard deviation. Model test–retest reliability, quantified using Krippendorff's alpha (nominal), was reported with 95% confidence intervals based on 1000 bootstrap iterations.^20^

## RESULTS

**FIGURE 2:**
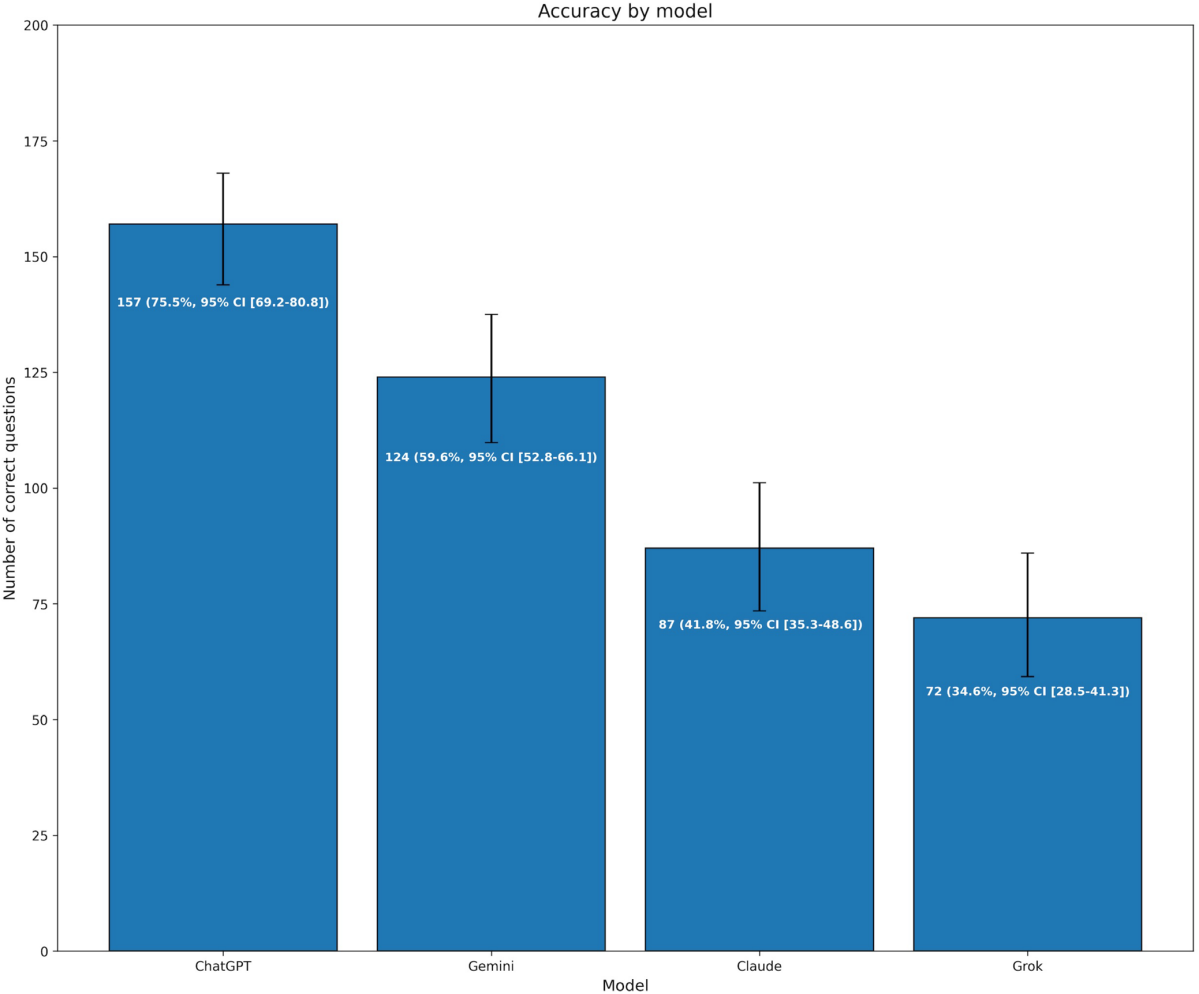
Overall model accuracy with 95% confidence intervals. Overall model accuracy for the four multimodal large language models (ChatGPT, Gemini, Claude, Grok), shown with corresponding 95% confidence intervals.

**FIGURE 3:**
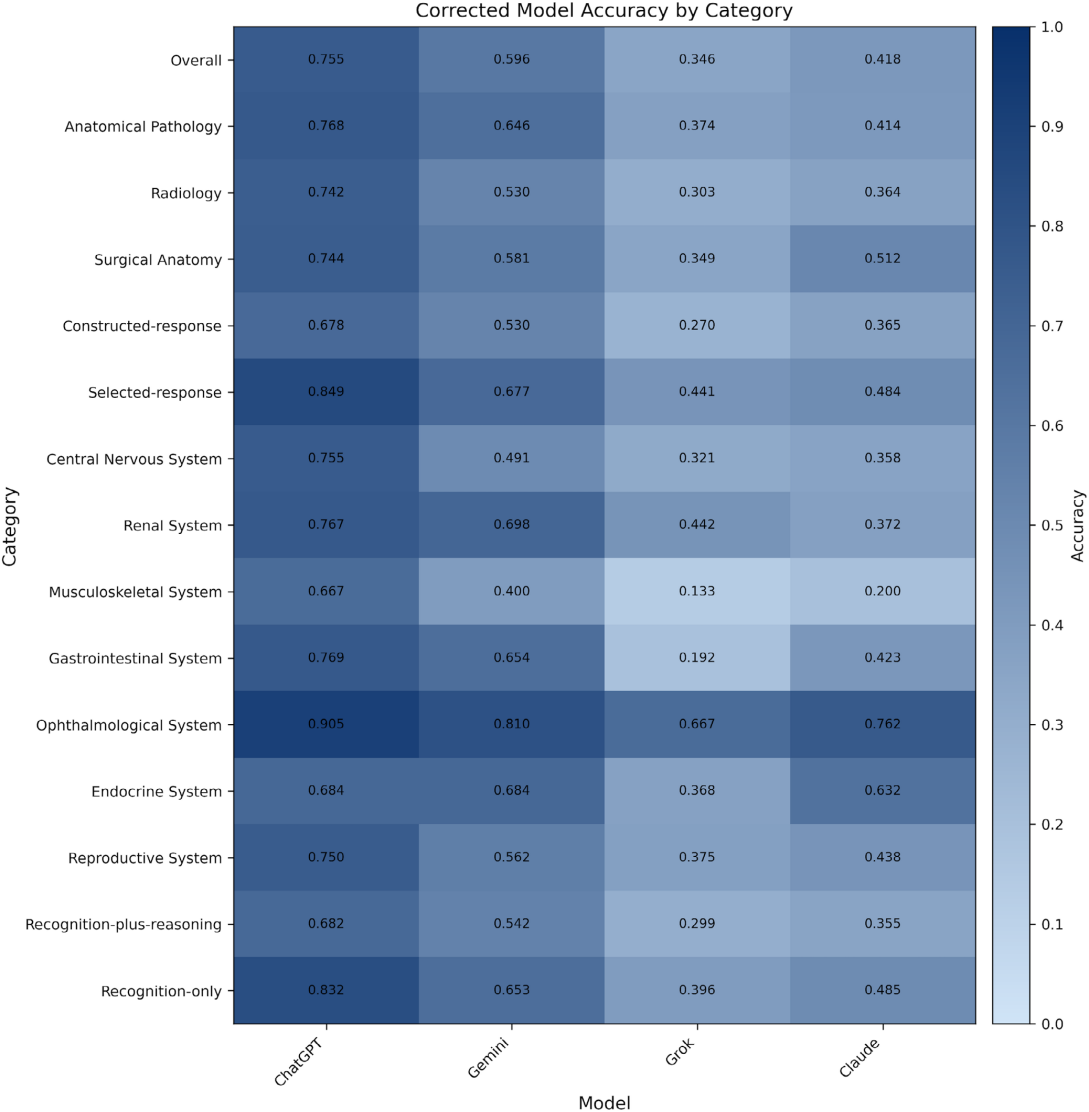
Heatmap of model accuracy by image modality, question format, organ system, and cognitive demand. Heatmap illustrating category‐level and subgroup‐level performance across image modality, question format, organ system, and cognitive demand for all four multimodal large language models.

The final bank comprised 208 image‐based questions (Table 1). Across 2496 model responses (4 models × 208 items × 3 attempts), inter‐rater agreement was almost perfect (Cohen's *κ* = 0.984; 95% CI [0.976–0.992]). Overall accuracy and subgroup analyses are summarized in Figure 2 and Table S1. Figure 3 provides a category‐level heatmap of model performance, and Tables 2 and 3 report statistical comparisons (*p*‐values and effect sizes).

**TABLE 1:** Characteristics of the image‐based examination question bank.

		Frequency	Percentage
Image modality	Anatomical pathology	99	47.6
	Radiology	66	31.7
	Surgical anatomy	43	20.7
Question format	Constructed‐response	115	55.3
	Selected‐response	93	44.7
Organ system	Central nervous system	53	25.5
	Renal system	43	20.7
	Musculoskeletal system	30	14.4
	Gastrointestinal system	26	12.5
	Ophthalmological system	21	10.1
	Endocrine system	19	9.1
	Reproductive system	16	7.7
Cognitive demand	Recognition‐plus‐reasoning	107	51.4
	Recognition‐only	101	48.6

*Note*: Characteristics of the 208‐item image‐based examination question bank, summarizing the distribution of image modality, question format, organ system, and cognitive demand.

**TABLE 2:** Cochran's *Q* test and pair‐wise McNemar's test *p* values.

Group type	Overall	ChatGPT vs. Gemini	ChatGPT vs. Claude	ChatGPT vs. Grok	Gemini vs. Claude	Gemini vs. Grok	Claude vs. Grok
Overall	<0.001*	<0.001*	<0.001*	<0.001*	<0.001*	<0.001*	0.064
Image modality							
Anatomical pathology	<0.001*	0.010*	<0.001*	<0.001*	<0.001*	<0.001*	0.540
Radiology	<0.001*	0.002*	<0.001*	<0.001*	0.022*	0.004*	0.522
Surgical anatomy	<0.001*	0.046*	0.004*	<0.001*	0.579	0.016*	0.070
Question format							
Constructed‐response	<0.001*	<0.001*	<0.001*	<0.001*	0.001*	<0.001*	0.046*
Selected‐response	<0.001*	0.002*	<0.001*	<0.001*	0.006*	<0.001*	0.596
Organ system							
Central nervous system	<0.001*	0.001*	<0.001*	<0.001*	0.169	0.052	0.789
Renal system	<0.001*	0.450	<0.001*	0.001*	0.002*	0.022*	0.628
Musculoskeletal system	<0.001*	0.013*	0.001*	<0.001*	0.077	0.013*	0.617
Gastrointestinal system	<0.001*	0.248	0.008*	<0.001*	0.114	0.001*	0.077
Ophthalmological system	0.205	0.617	0.248	0.131	1.000	0.505	0.617
Endocrine system	0.017*	0.617	1.000	0.041*	1.000	0.041*	0.131
Reproductive system	0.015*	0.248	0.074	0.041*	0.617	0.248	1.000
Cognitive demand							
Recognition‐plus‐reasoning	<0.001*	0.004*	<0.001*	<0.001*	0.001*	<0.001*	0.307
Recognition‐only	<0.001*	<0.001*	<0.001*	<0.001*	0.007*	<0.001*	0.164

*Note*: Statistical comparisons of model performance using Cochran's *Q* test and pairwise McNemar's tests (*p*‐values) across image modality, question format, organ system, and cognitive demand.

* *p* < 0.05 suggests statistical significance.

**TABLE 3:** Cochran's *Q* test and pairwise McNemar's test effect sizes.

Group type	Overall	ChatGPT vs. Gemini	ChatGPT vs. Claude	ChatGPT vs. Grok	Gemini vs. Claude	Gemini vs. Grok	Claude vs. Grok
Overall	0.449	0.159	0.337	0.409	0.178	0.250	0.072
Image modality							
Anatomical pathology	0.459	0.121	0.354	0.394	0.232	0.273	0.040
Radiology	0.433	0.212	0.379	0.439	0.167	0.227	0.061
Surgical anatomy	0.461	0.163	0.233	0.395	0.070	0.233	0.163
Question format							
Constructed‐response	0.424	0.148	0.313	0.409	0.165	0.261	0.096
Selected‐response	0.477	0.172	0.366	0.409	0.194	0.237	0.043
Organ system							
Central nervous system	0.446	0.264	0.396	0.434	0.132	0.170	0.038
Renal system	0.468	0.070	0.395	0.326	0.326	0.256	0.070
Musculoskeletal system	0.424	0.267	0.467	0.533	0.200	0.267	0.067
Gastrointestinal system	0.599	0.115	0.346	0.577	0.231	0.462	0.231
Ophthalmological system	0.456	0.095	0.143	0.238	0.048	0.143	0.095
Endocrine system	0.587	0.000	0.053	0.316	0.053	0.316	0.263
Reproductive system	0.270	0.188	0.312	0.375	0.125	0.188	0.062
Cognitive demand							
Recognition‐plus‐reasoning	0.459	0.140	0.327	0.383	0.187	0.243	0.056
Recognition‐only	0.440	0.178	0.347	0.436	0.168	0.257	0.089

*Note*: Effect sizes corresponding to Cochran's *Q* test and pairwise McNemar's tests for model performance comparisons across image modality, question format, organ system, and cognitive demand.

### Composition of the image‐based question Bank

Nearly half of the items were anatomical pathology images (99/208, 47.6%), with the remainder split between radiology (66/208, 31.7%) and surgical anatomy (43/208, 20.7%) (Table 1). Constructed‐response questions accounted for 115/208 items (55.3%), and selected‐response questions accounted for 93/208 (44.7%). Questions most often targeted the central nervous (53/208, 25.5%) and renal (43/208, 20.7%) systems, followed by musculoskeletal (30/208, 14.4%), gastrointestinal (26/208, 12.5%), ophthalmological (21/208, 10.1%), endocrine (19/208, 9.1%), and reproductive (16/208, 7.7%) systems. Cognitive demands were evenly distributed, comprising 107/208 recognition‐plus‐reasoning items (51.4%) and 101/208 recognition‐only items (48.6%).

### Overall model performance

Overall accuracy differed significantly between models (*p* < 0.001; Cramér's *V* = 0.449) (Figure 2; Tables 2, 3 and S1). ChatGPT‐5.1 achieved the highest accuracy, correctly answering 157/208 items (75.5%, 95% CI [69.2–80.8]), followed by Gemini‐2.5 with 124/208 (59.6%, 95% CI [52.8–66.1]). Claude Sonnet‐4.5 and Grok‐4 performed substantially worse, with 87/208 (41.8%, 95% CI [35.3–48.6]) and 72/208 (34.6%, 95% CI [28.5–41.3]) correct, respectively. Pairwise McNemar comparisons showed that ChatGPT‐5.1 outperformed all other models (all *p* < 0.001), with effect sizes ranging from 0.159 to 0.409. Gemini‐2.5 also exceeded both Claude Sonnet‐4.5 and Grok‐4 (*p* < 0.001), whereas the difference between Claude and Grok was not statistically significant (*p* = 0.064).

### Image modality

Marked inter‐model differences were observed across anatomical pathology, radiology, and surgical anatomy images (all *p* < 0.001) (Figure 3; Tables 2 and S1). Across all three modalities, ChatGPT‐5.1 consistently achieved the highest accuracy, followed by Gemini‐2.5, with Claude Sonnet‐4.5 and Grok‐4 performing substantially worse. Most pairwise contrasts were significant except those between Claude Sonnet‐4.5 vs. Grok‐4.

### Question format

Model accuracy differed notably by question format, but the inter‐model hierarchy remained consistent (ChatGPT > Gemini > Claude ≈ Grok; Figure 3; Tables 2 and S1). For constructed‐response items, accuracy was 67.8% for ChatGPT‐5.1, 53.0% for Gemini‐2.5, 36.5% for Claude Sonnet‐4.5, and 27.0% for Grok‐4. For selected‐response items, accuracy improved for all models: ChatGPT‐5.1 84.9%, Gemini‐2.5 67.7%, Claude Sonnet‐4.5 48.4%, and Grok‐4 44.1%. All pairwise differences were significant except Claude Sonnet‐4.5 vs. Grok‐4 in selected‐response items (*p* = 0.596).

### Organ system

Performance varied across organ systems but consistently favored ChatGPT‐5.1 (Figure 3; Tables 2 and S1). The largest between‐model differences were observed in musculoskeletal items, where ChatGPT‐5.1 achieved 66.7% accuracy compared with substantially lower performance by the other models (≤40%). In contrast, ophthalmology items showed uniformly high accuracy across all models, with no significant inter‐model differences. Renal and endocrine items demonstrated modest separation, with ChatGPT‐5.1 and Gemini‐2.5 performing similarly and both exceeding Claude Sonnet‐4.5 and Grok‐4.

### Cognitive demand

All models performed better on recognition‐only items than on recognition‐plus‐reasoning items. For recognition‐plus‐reasoning, accuracies were ChatGPT‐5.1 68.2%, Gemini‐2.5 54.2%, Claude Sonnet‐4.5 35.5%, and Grok‐4 29.9%; all contrasts were significant except Claude versus Grok (*p* = 0.307) (Figure 3; Tables 2 and S1). For recognition‐only, accuracies were ChatGPT‐5.1 83.2%, Gemini‐2.5 65.3%, Claude Sonnet‐4.5 48.5%, and Grok‐4 39.6%. All differences were significant except Claude Sonnet‐4.5 versus Grok‐4 (*p* = 0.164).

### Between‐model performance difference (BMPD), inter‐model variability (IMV), and test–retest reliability

A substantial between‐model performance difference was observed across items. The mean BMPD was 0.545 (SD = 0.451), indicating that, for many items, accuracy differed markedly between the highest‐ and lowest‐performing models. IMV was also notable, with a mean of 0.128 (SD = 0.123), consistent with meaningful dispersion in accuracy across models and identifying a subset of visually complex items that elicited pronounced inter‐model divergence.

Test–retest reliability differed between models. ChatGPT showed the highest agreement across repeated runs (Krippendorff's *α* = 0.868, 95% CI [0.798–0.931]). Grok demonstrated slightly lower agreement (*α* = 0.802, 95% CI [0.729–0.862]), while Gemini (*α* = 0.793, 95% CI [0.719–0.858]) and Claude (*α* = 0.789, 95% CI [0.718–0.852]) showed comparable levels of agreement. These findings suggest that multimodal LLMs generally have similar test–retest reliability, despite clear differences in accuracy.

## DISCUSSION

This study shows that, even in late 2025, leading multimodal LLMs do not yet reach the level of accuracy and reliability needed to support image‐based learning without supervision in medical education. All models exhibited persistent errors despite the use of high‐quality images and unambiguous marking keys (error rate 24.5%–65.4% across models). This finding is consistent with recent evaluations of anatomy education using commercial multimodal systems to interpret unlabeled anatomical images.^24^

### Interpretation of model performance

Across the 208 image‐based questions, we observed a consistent hierarchy: ChatGPT > Gemini > Claude ≈ Grok. This pattern aligns with other comparative studies in which GPT‐based systems generally outperform contemporary competitors on visual neuroanatomy questions and diagnostic radiology.^25^, ^26^, ^27^ However, absolute accuracy remained modest. In our study, ChatGPT‐5.1 correctly answered around three‐quarters of questions overall; Gemini‐2.5's accuracy rarely exceeded 65% in any domain; and Claude Sonnet‐4.5 and Grok‐4 consistently answered fewer than half of the items correctly. Compared with recent anatomy education evaluations of multimodal LLMs interpreting unlabeled anatomical images, which reported closer to two‐thirds correct, these results suggest a modest improvement, likely reflecting continued model progression.^24^

Performance varied by organ system in clinically intuitive ways. All models performed particularly well in ophthalmology, where retinal and slit‐lamp images are visually distinctive, but struggled more in musculoskeletal, gastrointestinal, and central nervous system items that required discriminating subtle morphological differences or integrating multiple features.

Similar domain‐specific variability, stronger performance in well‐represented domains (e.g., common radiology patterns), and weaker performance in specialized anatomical pathology or cross‐sectional anatomy, has been reported elsewhere.^23^, ^28^ These patterns probably reflect the distribution of training data and the fact that current general‐purpose multimodal LLMs are tuned for broad web imagery rather than deliberately balanced across medical specialties.

The contrast between recognition‐only and recognition‐plus‐reasoning items is striking. For all four models, accuracy on recognition‐only questions exceeded that on recognition‐plus‐reasoning questions by 10–15 percentage points. Our cognitive‐demand stratification, therefore, highlights an important limitation for educational use: models are relatively competent at “what is this structure?” but systematically weaker at “what common complications are seen in the pathology shown in the picture?” This pattern aligns with prior research indicating that current AI models perform better at feature‐label mapping than at relational or causal reasoning, and that errors in early image identification can subsequently propagate into downstream reasoning failures.^24^, ^29^, ^30^

The superiority of selected‐response over constructed‐response items for every model is also notable. Providing answer options likely reduces the cognitive load and allows models to leverage textual co‐occurrence statistics rather than relying solely on internal visual representations. Similar option‐related boosts in performance have been observed in USMLE‐style text‐only studies, where GPT‐4's scores drop when answer choices are removed.^4^, ^31^ For learners, this means that AI may appear more capable in multiple‐choice settings than in open‐ended interpretation tasks, potentially fostering an illusion of competence that does not translate to clinically realistic reasoning.

### Implications for medical education

Medical curricula require students to master both declarative knowledge and pattern‐based visual expertise.^9^, ^10^ While the former can often be acquired through self‐directed study supported by text‐based LLMs, visual skills such as reading radiographs, recognizing histopathological patterns, or localizing lesions on neuroimaging typically require guided exposure, expert feedback, and progressive refinement over years of training. Textbooks and atlases, though essential, are rarely sufficient on their own. Our findings suggest that current general‐purpose multimodal LLMs are not yet suitable as authoritative pedagogical resources for teaching image‐based medicine. Even in a relatively controlled environment (with high‐quality images, unambiguous single‐best answers, and a requirement to use only visual features), no model approached the reliability of experienced educators.

Despite their existing limitations, LLM tools are already deeply embedded in students' daily study routines. Learners increasingly turn to ChatGPT‐like systems to check answers, generate explanations, and rehearse clinical reasoning.^1^, ^2^, ^3^ In text‐based domains, this may be relatively safe if outputs are critically appraised against other sources. In image‐intensive domains, however, our data show that models will confidently provide wrong answers for a substantial minority of questions, with instability across attempts further complicating self‐assessment. For novices, who lack robust internal schemas to detect such errors, uncritical reliance on AI usage risks entrenching misconceptions and giving a false sense of mastery.

For medical students, multimodal LLMs are best viewed as adjunctive study partners rather than authoritative answer keys. When working with images, students should: (1) first attempt independent interpretation, (2) use the model primarily to obtain descriptive scaffolding, such as a stepwise description of salient features, rather than to shortcut to a diagnosis, and (3) cross‐check AI outputs against more traditional authoritative resources or educator feedback, especially when the answer diverges from their own reasoning. Repeating a question and observing whether the model's answer is stable can itself be a useful learning exercise; unstable outputs should be treated as a signal to seek human guidance rather than as an invitation to average across attempts.

For educators, these findings argue against a blanket prohibition of generative AI but strongly support structured, supervised integration. Multimodal LLMs might be used to rapidly draft low‐stakes practice items, generate alternative verbal explanations of classic imaging findings, or support case‐based discussions in which students critique and correct AI‐generated interpretations. However, any deployment should follow emerging ethical and assessment frameworks for AI in health professions education, including explicit oversight, alignment with learning outcomes, and clear communication of limitations to learners. High‐stakes assessments, whether formative decisions about progression or summative examinations, should remain anchored in human‐marked work and validated item banks rather than AI‐generated judgments.

### Limitations

This study has several limitations. First, the question bank was drawn from a single medical school. While the items were aligned in difficulty with the program's summative assessments, they may not reflect the full breadth of curricula, anatomical emphases, or assessment formats used elsewhere, which may limit generalizability beyond this institutional context.

Second, our design deliberately removed clinical vignettes and other textual cues to isolate pure visual reasoning. In practice, however, clinicians and students interpret images within a rich clinical context, and prior work shows that providing such context can substantially improve model accuracy.^16^ The performance estimates reported here should therefore be viewed as a lower bound rather than a comprehensive assessment of multimodal diagnostic capability.

Third, we evaluated only one prompt template and one configuration per model; alternative prompts, temperature settings, or API‐based control might yield different results, although prior work suggests that prompt effects on image interpretation are modest compared with those of the underlying model architecture.^32^, ^33^

Fourth, we analyzed only final answers and did not assess the quality, explainability, or potential educational value of model rationales, because explanations were suppressed to prevent inadvertent use of textual cues. Explanatory capacity may still hold educational value even when final answers are occasionally wrong.

### Future directions

Future research should focus on continued, standardized evaluation of multimodal LLMs on authentic image‐based assessment items, including assessment of accuracy, error patterns, and output stability across model versions. For factual, non‐ambiguous image‐based tasks, performance at or near the expert level should be expected, and evaluation frameworks should be able to verify whether these standards are met. AI models and their training data are evolving quickly; within months of our testing, updated versions may already exhibit different performance profiles. Regular re‐evaluation using similar STAGER‐compliant protocols will therefore be essential.

## CONCLUSIONS

In summary, this evaluation of four frontier multimodal LLMs on authentic image‐based medical school examination questions shows that, although ChatGPT currently leads its peers, no model yet delivers the accuracy and reliability required for unsupervised use as a primary teacher or assessor of image‐based competencies. Given the centrality of visual diagnostic reasoning to medical practice and the intrinsic difficulty of acquiring such skills, multimodal LLMs should presently be integrated into medical education as supervised, critically appraised adjuncts rather than trusted authorities. Continuous, methodologically rigorous benchmarking, paired with explicit guidance for learners and educators, will be essential as these rapidly evolving systems are increasingly woven into the fabric of medical training.

## AUTHOR CONTRIBUTIONS

**Josiah Cheng:** Investigation; validation; writing – review and editing; data curation. **Vinod Gopalan:** Conceptualization; writing – review and editing; project administration; supervision; data curation; resources; validation; methodology; investigation. **Ming Lu:** Conceptualization; investigation; writing – original draft; methodology; software; formal analysis; project administration; data curation; validation; writing – review and editing.

## FUNDING INFORMATION

No external funding was received.

## CONFLICT OF INTEREST STATEMENT

The authors declare no competing interests.

## ETHICS STATEMENT

This study involved only de‐identified educational assessment materials and therefore did not require human research ethics approval.

## PERMISSIONS

All images originated from the internal teaching and assessment resources of the Griffith University MD program; no third‐party copyrighted material was used.

The examination questions and marking keys used in this study were originally developed by the Griffith University histopathology education team led by Vinod Gopalan. Permission has been granted for their use in this research.

## Supporting information


**Table S1.** Model accuracy by image modality, question format, organ system, and cognitive demand.

## Data Availability

Examination items cannot be shared due to assessment security; aggregated data are available on reasonable request.

## References

[1] Hu N , Jiang XQ , Wang YD , Kang YM , Xia Z , Chen HH , et al. Status and perceptions of ChatGPT utilization among medical students: a survey‐based study. BMC Med Educ. 2025 Jun 4;25(1):831. 40468340 10.1186/s12909-025-07438-7 PMC12135314

[2] Tran C , Hryciw BN , Moore SW , Chaput A , Seely AJE . Perceptions and use of generative artificial intelligence in medical students: a multicenter survey. J Med Educ Curric Dev [Internet]. 2025 Oct [cited 2025 Nov 14];12:23821205251391969. Available from: https://pmc.ncbi.nlm.nih.gov/articles/PMC12576227/ 41181167 10.1177/23821205251391969 PMC12576227

[3] Tajima H , Kasai H , Shikino K , Shimizu I , Ito S . Perceptions and intentions to use generative artificial intelligence among first‐year medical students in Japan: a cross‐sectional survey study (preprint). JMIR Med Educ. 2025 May 16;11:e77552. 41232013 10.2196/77552 PMC12614389

[4] Liu M , Okuhara T , Chang X , Shirabe R , Nishiie Y , Okada H , et al. Performance of ChatGPT across different versions in medical licensing examinations worldwide: a systematic review and meta‐analysis (preprint). J Med Internet Res [Internet]. 2024 Jul 25;26:e60807. Available from: https://pubmed.ncbi.nlm.nih.gov/39052324/ 39052324 10.2196/60807 PMC11310649

[5] Meyer A , Riese J , Streichert T . Comparison of the performance of GPT‐3.5 and GPT‐4 with that of medical students on the written German medical licensing examination: observational study. JMIR Med Educ [Internet]. 2024 Feb 8;10:e50965. Available from: https://pubmed.ncbi.nlm.nih.gov/38329802/ 38329802 10.2196/50965 PMC10884900

[6] Bicknell BT , Butler D , Whalen S , Ricks J , Dixon CJ , Clark AB , et al. ChatGPT‐4 Omni performance in USMLE disciplines and clinical skills: comparative analysis. JMIR Med Educ [Internet]. 2024 Nov 6;10:e63430. Available from: https://www.sciencedirect.com/org/science/article/pii/S2369376224001338 39504445 10.2196/63430 PMC11611793

[7] Luo D , Liu M , Yu R , Liu Y , Jiang W , Fan Q , et al. Evaluating the performance of GPT‐3.5, GPT‐4, and GPT‐4o in the Chinese National Medical Licensing Examination. Sci Rep [Internet]. 2025 Apr 23 [cited 2025 Jun 2];15(1):14119. Available from: https://www.nature.com/articles/s41598‐025‐98949‐2 40269046 10.1038/s41598-025-98949-2 PMC12018924

[8] Wu YC , Wu YC , Chang YC , Yu CY , Wu CL , Sung WW . Advancing medical AI: GPT‐4 and GPT‐4o surpass GPT‐3.5 in Taiwanese medical licensing exams. PLoS One [Internet]. 2025;20(6):e0324841. Available from: https://pubmed.ncbi.nlm.nih.gov/40465748/ 40465748 10.1371/journal.pone.0324841 PMC12136359

[9] Frenk J , Chen L , Bhutta ZA , Cohen J , Crisp N , Evans T , et al. Health professionals for a new century: transforming education to strengthen health Systems in an Interdependent World. Lancet. 2010 Dec;376(9756):1923–1958. 21112623 10.1016/S0140-6736(10)61854-5

[10] Basic medical education WFME global standards for quality improvement: The 2020 Revision [Internet]. Basic Medical Education. World Federation for Medical Education (WFME); 2020 [cited 2025 Nov 22]. Available from: https://wfme.org/standards/bme/

[11] Yin S , Fu C , Zhao S , Li K , Sun X , Xu T , et al. A survey on multimodal large language models. Natl Sci Rev. 2024 Nov 12;11(12):nwae403. 39679213 10.1093/nsr/nwae403 PMC11645129

[12] Wang J , Jiang H , Liu YH , Ma CY , Zhang X , Pan Y , et al. A comprehensive review of multimodal large language models: performance and challenges across different tasks. arXivorg [Internet]. 2024 [cited 2024 Dec 12];18(9):2408.01319. Available from: https://www.semanticscholar.org/paper/A‐Comprehensive‐Review‐of‐Multimodal‐Large‐Language‐Wang‐Jiang/5ea16dfb5a44a97a8e93177e7e588cc7fb9e5996

[13] Yang Z , Yao Z , Tasmin M , Vashisht P , Jang WS , Ouyang F , et al. Unveiling GPT‐4V's hidden challenges behind high accuracy on USMLE questions: observational study. J Med Internet Res. 2025 Feb 7;27:e65146. 39919278 10.2196/65146 PMC11845889

[14] Yang X , Chen W . The performance of ChatGPT on medical image‐based assessments and implications for medical education. BMC Med Educ. 2025 Aug 23;25(1):1192. 40849473 10.1186/s12909-025-07752-0 PMC12374324

[15] Gajjar AA , Valluri H , Prabhala T , Custozzo A , Boulos AS , Dalfino JC , et al. Evaluating the performance of ChatGPT‐4o vision capabilities on image‐based USMLE step 1, step 2, and step 3 examination questions. bioRxiv (Cold Spring Harbor Laboratory). 2024 Jun 19.

[16] Atakır K , Işın K , Taş A , Önder H . Diagnostic accuracy and consistency of ChatGPT‐4o in radiology: influence of image, clinical data, and answer options on performance. Diagn Interv Radiol. 2025 Sep 22. Available from: 10.4274/dir.2025.253460 PMC13530496 40977631

[17] Chen J , Zhu L , Mou W , Lin A , Zeng D , Qi C , et al. STAGER checklist: standardized testing and assessment guidelines for evaluating generative artificial intelligence reliability. iMetaOmics. 2024 Jul 2;1(1):e7. 41675544 10.1002/imo2.7 PMC12806434

[18] Tavakol M , O'Brien D . Psychometrics for physicians: everything a clinician needs to know about assessments in medical education. Int J Med Educ. 2022 Apr 22;13:100–106. 35462355 10.5116/ijme.625f.bfb1 PMC9902176

[19] Haase J , Hanel PHP , Pokutta S . Has the creativity of large‐language models peaked?: an analysis of inter‐ and intra‐LLM variability. J Creat. 2025 Nov 8;35(3):100113.

[20] Krippendorff K . Reliability in content analysis: some common misconceptions and recommendations. Hum Commun Res. 2004 Jul 1;30(3):411–433.

[21] Berry KJ , Johnston JE , Mielke PW . An alternative measure of effect size for Cochran's *Q* test for related proportions. Percept Mot Skills. 2007 Jun;104(3_suppl):1236–1242. 17879655 10.2466/pms.104.4.1236-1242

[22] McHugh ML . Cramér's V coefficient. The SAGE encyclopedia of educational research, measurement, and evaluation. Thousand Oaks, CA: SAGE publications; 2018.

[23] Zhao YD , Rahardja D . Estimation of the common risk difference in stratified paired binary data with homogeneous stratum effect. J Biopharm Stat. 2013 Jun 20;23(4):848–855. 23786205 10.1080/10543406.2013.789888

[24] Hyeamang LJ , Sekhar TC , Rush E , Beresheim AC , Cheverko CM , Brooks WS , et al. AI's ability to interpret unlabeled anatomy images and supplement educational research as an AI rater. Anat Sci Educ. 2025 Jul 11;18(10):1102–1113. 40646660 10.1002/ase.70074 PMC12511656

[25] Güneş YC , Ülkir M . Comparative performance evaluation of multimodal large language models, radiologist, and anatomist in visual neuroanatomy questions. Uludağ Univ Tip Fak Derg. 2025 Jan 12;50(3):551–556.

[26] Sun SH , Chen K , Anavim S , Phillipi M , Yeh L , Huynh K , et al. Large language models with vision on diagnostic radiology board exam style questions. Acad Radiol. 2024 Dec 1;313(1):e240609. 10.1016/j.acra.2024.11.028 39632215

[27] Suh PS , Shim WH , Suh CH , Heo H , Park CR , Eom HJ , et al. Comparing diagnostic accuracy of radiologists versus GPT‐4V and Gemini pro vision using image inputs from diagnosis please cases. Radiology. 2024 Jul 1;312(1):e240273. 38980179 10.1148/radiol.240273

[28] Sun Y , Wu H , Zhu C , Zheng S , Chen Q , Zhang K , et al. PathMMU: a massive multimodal expert‐level benchmark for understanding and reasoning in pathology. arXiv (Cornell University). 2024 Jan 29.

[29] Bazi Y , Rahhal A , Bashmal L , Zuair M . Vision–language model for visual question answering in medical imagery. Bioengineering. 2023 Mar 20;10(3):380. 36978771 10.3390/bioengineering10030380 PMC10045796

[30] Kim J , Podlasek A , Shidara K , Liu F , Alaa A , Bernardo D . Limitations of large language models in clinical problem‐solving arising from inflexible reasoning. Sci Rep. 2025 Nov 11;15(1):39426. 41219270 10.1038/s41598-025-22940-0 PMC12606185

[31] Casals‐Farre O , Baskaran R , Singh A , Kaur H , Hoque TU , de Almeida A , et al. Assessing ChatGPT 4.0's capabilities in the United Kingdom medical licensing examination (UKMLA): a robust categorical analysis. Sci Rep. 2025 Apr 15;15(1):13031. 40234701 10.1038/s41598-025-97327-2 PMC12000555

[32] Jeon S , Kim HG . A comparative evaluation of chain‐of‐thought‐based prompt engineering techniques for medical question answering. Comput Biol Med. 2025 Jul 1;196(Pt A):110614. 40602316 10.1016/j.compbiomed.2025.110614

[33] Chen B , Zhang Z , Langrené N , Zhu S . Unleashing the potential of prompt engineering for large language models. Patterns [Internet]. 2025 May;6(6):101260. Available from: https://www.cell.com/patterns/fulltext/S2666‐3899(25)00108‐4 40575123 10.1016/j.patter.2025.101260 PMC12191768

