# Supplementary material for: Performance of multimodal large language models on image‐based surgical anatomy, anatomical pathology, and radiology questions
Source: Anat Sci Educ. 2026 May 13;19(8):1288–99. doi: 10.1002/ase.70256 (PMC13440719; doi:10.1002/ase.70256)
Supplement: Supplementary file 1 [file ASE-19-1288-s001.docx]

|  |  | No. of Questions | **ChatGPT** | | **Gemini** | | **Grok** | | **Claude** | |
| --- | --- | --- | --- | --- | --- | --- | --- | --- | --- | --- |
|  |  |  | No. of Correct responses | Accuracy (95% CI) | No. of Correct responses | Accuracy (95% CI) | No. of Correct responses | Accuracy (95% CI) | No. of Correct responses | Accuracy (95% CI) |
| Image Modality | Anatomical Pathology | 99 | 76 | 0.768 (0.675–0.840) | 64 | 0.646 (0.548–0.734) | 37 | 0.374 (0.285–0.472) | 41 | 0.414 (0.322–0.513) |
|  | Radiology | 66 | 49 | 0.742 (0.626–0.833) | 35 | 0.530 (0.412–0.646) | 20 | 0.303 (0.206–0.422) | 24 | 0.364 (0.258–0.484) |
|  | Surgical Anatomy | 43 | 32 | 0.744 (0.598–0.851) | 25 | 0.581 (0.433–0.716) | 15 | 0.349 (0.224–0.498) | 22 | 0.512 (0.368–0.654) |
| Question Format | Constructed-response | 115 | 78 | 0.678 (0.588–0.757) | 61 | 0.530 (0.440–0.619) | 31 | 0.270 (0.197–0.357) | 42 | 0.365 (0.283–0.456) |
|  | Selected-response | 93 | 79 | 0.849 (0.763–0.908) | 63 | 0.677 (0.577–0.764) | 41 | 0.441 (0.344–0.542) | 45 | 0.484 (0.385–0.584) |
| Organ System | Central Nervous System | 53 | 40 | 0.755 (0.624–0.851) | 26 | 0.491 (0.361–0.621) | 17 | 0.321 (0.211–0.455) | 19 | 0.358 (0.243–0.493) |
|  | Renal System | 43 | 33 | 0.767 (0.623–0.868) | 30 | 0.698 (0.549–0.814) | 19 | 0.442 (0.304–0.589) | 16 | 0.372 (0.244–0.521) |
|  | Musculoskeletal System | 30 | 20 | 0.667 (0.488–0.808) | 12 | 0.400 (0.246–0.577) | 4 | 0.133 (0.053–0.297) | 6 | 0.200 (0.095–0.373) |
|  | Gastrointestinal System | 26 | 20 | 0.769 (0.579–0.890) | 17 | 0.654 (0.462–0.806) | 5 | 0.192 (0.085–0.379) | 11 | 0.423 (0.255–0.611) |
|  | Ophthalmological System | 21 | 19 | 0.905 (0.711–0.973) | 17 | 0.810 (0.600–0.923) | 14 | 0.667 (0.454–0.828) | 16 | 0.762 (0.549–0.894) |
|  | Endocrine System | 19 | 13 | 0.684 (0.460–0.846) | 13 | 0.684 (0.460–0.846) | 7 | 0.368 (0.191–0.590) | 12 | 0.632 (0.410–0.809) |
|  | Reproductive System | 16 | 12 | 0.750 (0.505–0.898) | 9 | 0.562 (0.332–0.769) | 6 | 0.375 (0.185–0.614) | 7 | 0.438 (0.231–0.668) |
| Cognitive Demand | Recognition-plus-reasoning | 107 | 73 | 0.682 (0.589–0.763) | 58 | 0.542 (0.448–0.633) | 32 | 0.299 (0.221–0.392) | 38 | 0.355 (0.271–0.449) |
|  | Recognition-only | 101 | 84 | 0.832 (0.747–0.892) | 66 | 0.653 (0.557–0.739) | 40 | 0.396 (0.306–0.494) | 49 | 0.485 (0.390–0.581) |

Supplementary Table 1. Model accuracy by image modality, question format, organ system, and cognitive demand
